# Membrane Hsp70—A Novel Target for the Isolation of Circulating Tumor Cells After Epithelial-to-Mesenchymal Transition

**DOI:** 10.3389/fonc.2018.00497

**Published:** 2018-11-01

**Authors:** Stephanie Breuninger, Stefan Stangl, Caroline Werner, Wolfgang Sievert, Dominik Lobinger, Gemma A. Foulds, Sarah Wagner, Anja Pickhard, Guido Piontek, Konrad Kokowski, Alan G. Pockley, Gabriele Multhoff

**Affiliations:** ^1^Center for Translational Cancer Research TU München (TranslaTUM), Klinikum rechts der Isar, TUM, Munich, Germany; ^2^John van Geest Cancer Research Centre, College of Science and Technology, Nottingham Trent University, Nottingham, United Kingdom; ^3^Department of Otolaryngology Head and Neck Surgery, Klinikum rechts der Isar, TUM, Munich, Germany; ^4^Department of Pneumology and Pneumologic Oncology, Klinikum Bogenhausen, Munich, Germany

**Keywords:** circulating tumor cells (CTCs), membrane Hsp70, cmHsp70.1 antibody, EpCAM (CD326), epithelial-to-mesenchymal transition (EMT)

## Abstract

The presence of circulating tumor cells (CTCs) in the peripheral blood is a pre-requisite for progression, invasion, and metastatic spread of cancer. Consequently, the enumeration and molecular characterization of CTCs from the peripheral blood of patients with solid tumors before, during and after treatment serves as a valuable tool for categorizing disease, evaluating prognosis and for predicting and monitoring therapeutic responsiveness. Many of the techniques for isolating CTCs are based on the expression of epithelial cell surface adhesion molecule (EpCAM, CD326) on tumor cells. However, the transition of adherent epithelial cells to migratory mesenchymal cells (epithelial-to-mesenchymal transition, EMT)—an essential element of the metastatic process—is frequently associated with a loss of expression of epithelial cell markers, including EpCAM. A highly relevant proportion of mesenchymal CTCs cannot therefore be isolated using techniques that are based on the “capture” of cells expressing EpCAM. Herein, we provide evidence that a monoclonal antibody (mAb) directed against a membrane-bound form of Hsp70 (mHsp70)—cmHsp70.1—can be used for the isolation of viable CTCs from peripheral blood of tumor patients of different entities in a more quantitative manner. In contrast to EpCAM, the expression of mHsp70 remains stably upregulated on migratory, mesenchymal CTCs, metastases and cells that have been triggered to undergo EMT. Therefore, we propose that approaches for isolating CTCs based on the capture of cells that express mHsp70 using the cmHsp70.1 mAb are superior to those based on EpCAM expression.

## Introduction

The malignancy of solid tumors is determined by the heterogeneity of the individual tumor (1) and its capacity to disseminate from the primary tumor to secondary sites via the blood stream. This process involves fundamental activities including invasion of the extracellular matrix and surrounding stroma, migration, evasion from the vasculature, proliferation, and the avoidance of anoikis (a form of programmed cell death that occurs in anchorage-dependent cells when they detach from the surrounding extracellular matrix) (2). Due to multiple selection steps, primary tumors and corresponding metastases often differ in molecular markers and pathways (3). Interrogating the biology of CTCs and also circulating tumor cell aggregates, which are generally considered as precursors of metastasis (4), is likely to provide invaluable insights into the biology of the disease and the prevalence and heterogeneity of future metastasis and thereby help to predict outcome and identify new tumor biomarkers that could be targeted by individualized anti-tumor therapies (5). Isolation, enumeration and the molecular characterization of CTCs could therefore bridge the “missing link” between cancer biology and individualized/precision therapies. As a liquid biopsy, CTCs do not only provide an opportunity to identify therapeutic targets and resistance mechanisms and monitor therapeutic responsiveness, but can also provide prognostic information about the risk of developing metastatic relapse and tumor progression which underpin 90% of cancer-related deaths (6–8).

A major limitation to the application of CTC-based analysis is the rarity of this cell type in the peripheral blood. Typically, 1 ml of peripheral blood of patients with metastatic cancer contains <10 CTCs (9–11). As a consequence, different strategies including filtration- (12, 13), microfluidic chip- (14–17), PCR- (18–20), and flow cytometry-based techniques (21) have been developed to enrich, separate and differentiate CTCs in the peripheral blood from the vastly more prominent hematopoietic cell population (1 CTC in 10^6^-10^8^ hematopoietic cells). Although a variety of biomarkers such as CD44, CD133, CD47, cMET, EGFR, and immune checkpoint inhibitors are heterogeneously expressed on CTCs derived from different tumor entities, the most common techniques for the *ex vivo* separation of CTCs from peripheral blood are based on the capturing of cells using antibodies directed against cell surface expressed EpCAM (CD326) (22–26). The CellSearch® system (27)—the FDA-approved “gold standard”—combines a magnetic separation technique based on EpCAM antibody-coated particles with subsequent cytokeratin (CK) staining and a microscopic analysis of the isolated cells (22). Another limitation of most *ex vivo* CTC isolation techniques is the relatively small blood sample volume (7.5 ml) which is used and the low numbers of CTCs that can be derived therefrom. To overcome these disadvantages of *ex vivo* CTC isolation, GILUPI GmbH (Potsdam, Germany) has developed an EpCAM antibody-coated CellCollector® system which involves the direct insertion of a stainless steel wire, functionalized with gold and a hydrogel coating that incorporates anti-EpCAM antibodies, into the blood stream via a standard venous cannula in the cubital veins for 30 min. During this period, CTCs can be captured from the entire peripheral blood compartment (several liters of blood) of a cancer patient. Subsequently, the captured viable cells can be stained whilst attached to the wire and analyzed by fluorescence microscopy (28) or expanded for further analysis. The number of CTCs captured by the CellCollector® system before and after therapy has been shown to be associated with prognosis and therapeutic responsiveness (11).

All the techniques described above rely on the cell surface expression of EpCAM and the lack of the leukocyte marker CD45 by CTCs. However, many studies have shown that the transition of the adherent epithelial cells to the migratory mesenchymal state which enables the motility and invasiveness of CTCs and their dissemination to distant sites is associated with a loss in the expression of classical epithelial cell markers, including EpCAM (29). Yu et al. demonstrated that benign and non-invasive tumor cells exclusively express epithelial antigens, whereas a subpopulation of invasive breast cancer cells express both epithelial and mesenchymal markers (30). Epithelial-to-mesenchymal transition (EMT) correlates with an increased migratory and metastatic potential of CTCs, invasiveness, poor overall survival and drug resistance (29, 30). It is therefore apparent that systems for isolating CTCs that rely only on the expression of epithelial markers by target cells are limited in their ability to detect CTCs arising after EMT.

The search for universal tumor markers has revealed that the major stress-inducible heat shock protein 70 (Hsp70) is frequently expressed on the plasma membrane of primary tumor cells and distant metastases (31). This membrane Hsp70 (mHsp70) positivity has been identified on a large variety of different primary tumor types such as breast, lung, head and neck, colorectal, pancreas, brain and hematological malignancies, but not on corresponding normal cells and tissues (32, 33). A comparison of the cell surface density of Hsp70 has also revealed higher intensities of mHsp70 on metastases compared to corresponding primary tumors in mouse and human models (33–36). This finding provides a first indication that the expression of mHsp70 might not be downregulated by EMT and that it could therefore serve as a useful target for the isolation of CTCs in the circulation that have undergone EMT. Given that our group has developed a unique mouse monoclonal antibody (mAb) termed cmHsp70.1 which specifically detects the membrane-bound form of Hsp70 on viable tumor cells (37), herein we determine the capacity of the cmHsp70.1 mAb to form the basis of improved bead- and wire-based CTC isolation techniques that exploit mHsp70 expression as a universal tumor-specific biomarker.

## Materials and methods

### Ethics, patient characteristics

Signed informed consent was obtained from all patients with squamous cell carcinoma of the head and neck (SCCHN) and non-small cell lung carcinoma (NSCLC) before EDTA blood (1–2 × 7.5 ml) was taken and the protocol was approved by the institutional ethical review board of the Klinikum rechts der Isar at the Technische Universität München (TUM). Eight patients with SCCHN in tumor stages pT1 to pT3, one patient with cancer of unknown primary (CUP) tumor of the head and neck, and three patients with advanced adenocarcinoma of the lung (NSCLC) were included into the study.

### Cell lines and culture

Studies undertaken at the Technische Universität München used the following human cell lines: breast cancer cell lines MDA-MB-231 (ATCC HTB-26), MCF7 (ATCC HTB-22), SK-BR-3 (ATCC HTB-30), and T47D (ATCC HTB-133), lung cancer cell lines EPLC-272H (DSMZ ACC-383), H1339 (DSMZ ACC-506), A549 (ATCC CCL-185), melanoma cell line LS174T (ATCC CL-188, kindly provided by Professor Marina Kreutz, University Hospital Regensburg, Germany), pancreatic cancer cell lines PANC-1 (ATCC CRL-1469), MIA PaCa-2 (ATCC CRL-1420), cervical cancer cell line HeLa (ATCC CCL-2), colon cancer cell line HCT-15 (ATCC CCL-225), brain cancer cell lines LN-229 (ATCC CRL-2611), U-87 (ATCC HTB-14), squamous cell carcinoma cell lines of the head and neck UP154, UD5 (kindly provided by Professor Anja Pickhard, Department of Otolaryngology Head and Neck Surgery, Klinikum rechts der Isar, TUM), prostate cancer cell line DU145 (ATCC HTB-81). Studies undertaken at Nottingham Trent University used the following human cell lines: breast cancer cell lines MDA-MB-231 (ATCC HTB-26), MCF7 (ATCC HTB-22), SK-BR-3 (ATCC HTB-30) and T47D (ATCC HTB-133), lung cancer (A549) and prostate cancer DU145 (ATCC HTB-81).

All cells were maintained under standard conditions (37°C, 95% v/v humidity, 5% v/v CO_2_) in appropriate cell culture medium. Cell line authentication was performed by DNA profiling using highly polymorphic short tandem repeats (DSMZ-Deutsche Sammlung von Mikroorganismen und Zellkulturen, Leipzig, Germany (TUM) and the ATCC Cell Line Authentication Service (NTU). Cells were passaged every 3–4 days and used in the exponential growth phase. Cell viability was confirmed prior to use using trypan blue dye exclusion (>95%). Cell cultures were routinely tested to ensure the absence of mycoplasma.

### Influence of TGFβ-induced epithelial-to-mesenchymal transition (EMT) on mHsp70 and EpCAM (CD326) expression

EMT was induced in DU145 prostate, UP154, and UD5 squamous cell carcinoma cells of the head and neck and A549 lung cancer cells by treatment with TGFβ (38). For this, after initial seeding, cells were allowed to adhere for 24 h, after which the medium was exchanged with medium containing 10 ng/ml TGFβ (PeproTech) every second day. Control cells were sham treated in standard cell culture medium. After 10 days of cell culture with regular media changes, cells were harvested and the expression of mHsp70 and EpCAM on viable cells was determined by 2-color flow cytometry, as detailed below.

Photomicroscopic views were taken from untreated and TGFβ (10 ng/ml) treated A549 cells on day 10, day 10 + 4 days recovery without TGFβ and day 10 + 7 days recovery without TGFβ.

### Influence of L-lactic-acid-induced epithelial-to-mesenchymal transition (EMT) on mHsp70 and EpCAM (CD326) expression

In addition to TGFβ, EMT was induced in A549 lung cancer cells, LS174T melanoma cells and UP154 and UD5 squamous cell carcinoma cells of the head and neck by a treatment with L-lactic-acid (Lac-Ac). For this, after initial seeding, cells were incubated with medium containing L-lactic-acid (Lac-Ac, 10 mM; pH 6.8). Up to a concentration of 20 mM, L-lactic-acid did not induce significant cell death in tumor cells. After 2 days, when cells reached a confluency of 70–80% cells were harvested and analyzed for the expression of EpCAM (CD326) and mHsp70 by flow cytometry. Control cells were sham treated with standard culture medium.

### Flow cytometric analysis of membrane Hsp70 (mHsp70) and EpCAM (CD326) expression

At the Technische Universität München, flow cytometric analysis for the expression of mHsp70 and EpCAM on tumor cell lines was undertaken using either an FITC-conjugated cmHsp70.1 mAb (multimmune GmbH, Munich, Germany) and a FITC-conjugated EpCAM (CD326, clone HEA125) and PE-conjugated EpCAM (CD326, clone G8.8) mAb (Acris GmbH, an OriGene Company, Herford, Germany). Briefly, cells (1 × 10^5^) were incubated with the relevant antibody for 30 min at 4°C, washed once and viable cells (propidium iodide negative) were analyzed using a FACSCalibur™ flow cytometer (BD Biosciences, Franklin Lakes, NJ, USA). An isotype-matched (IgG1) control antibody (BD Biosciences) was used to evaluate non-specific binding to cells.

At Nottingham Trent University, the co-expression of mHsp70 and EpCAM on A549 and DU145 cells that had been induced to undergo EMT by treatment with TGFβ was determined by incubating cells (1 × 10^5^) with a FITC-conjugated cmHsp70.1 mAb (multimmune GmbH), a PerCP-Vio770 conjugated EpCAM (CD326) mAb (Miltenyi Biotec, Clone REA764), and LIVE/DEAD™ Fixable Yellow Dead Cell Stain (Invitrogen) at 4°C for 30 min. Unstained cells, cells incubated with an isotype-matched control mAb and cells incubated with an FITC-conjugated mAb recognizing an epitope of Hsp70 which is inaccessible on the cell surface (clone C92, Stressgen Biotechnologies Corpn., Victoria, Canada) served as controls. After incubation, cells were washed once and the expression of mHsp70 and EpCAM (CD326) by viable cells was determined using a 3-laser, 10-color Beckman Coulter Gallios™ flow cytometer and Kaluza™ software.

### Collection of peripheral blood and isolation of peripheral blood mononuclear cells (PBMCs) using Ficoll

Blood was collected from healthy volunteers into K3 EDTA tubes (S-Monovette, Sarstedt, Nümbrecht, Germany) and mixed with at least an equal amount of RPMI 1640 (Sigma-Aldrich, St. Louis, USA) medium. For bead-based cell isolation experiments (see below), harvested tumor cells which had been washed once with phosphate-buffered saline (PBS) were added to the anticoagulated whole blood at the indicated concentrations. Peripheral blood mononuclear cells (PBMCs) and tumor cells were then isolated using a Ficoll-based density gradient lymphocyte separation medium (LSM 1077) (GE Healthcare, Chalfont St. Giles, UK). Following centrifugation at 850 g at room temperature (RT) for 20 min, PBMCs and tumor cells were carefully extracted from the interphase between the plasma and the separation solution. The cells were washed twice in medium. For further experiments (see below), cells were allowed to recover under rotation in 10% v/v fetal bovine serum (FBS, Sigma-Aldrich, St. Louis, USA) in PBS for 4 h.

### Cell isolation with magnetic dynabeads

Coupling the cmHsp70.1 mAb or EpCAM mAb (CD326, clone HE125) to magnetic Dynabeads™ (Thermo Fisher, Waltham, USA) was carried out according to the manufacturer's protocol. Dynabeads coupled with cmHsp70.1 mAb were washed and incubated either with tumor cells, a mixture of tumor cells and lymphocytes or EDTA blood (7.5 ml) of patients with squamous cell carcinoma of the head and neck (SCCHN) and non-small cell lung carcinoma (NSCLC) after removal of red blood cells for 10 min at 4°C under rotation. The tube was placed in a DynaMag™ magnet and beads were separated from the supernatant after a washing step. The isolated CTCs were counted and stained with DAPI (4′,6-diamidino-2-phenylindole), cytokeratin-Alexa 488, EpCAM-PE and cmHsp70.1-FITC mAb. Isolated CTCs from tumor patients were clonally seeded in 96-well round-bottom culture plates.

### Cell capture and analysis on the GILUPI CellCollector®

For determining the capacity of the GILUPI CellCollector® (GILUPI GmbH, Potsdam, Germany) to capture tumor cells, cells were either pre-stained with the Vybrant™ CFDA Cell Tracer Kit (Thermo Fisher, Waltham, USA) or stained on the wire after cell capture. 2 ml reaction tubes (Sarstedt, Nürmbrecht, Germany) were blocked with 3% w/v bovine serum albumin (BSA, Sigma-Aldrich, St. Louis, USA) in PBS (Sigma-Aldrich, St. Louis, USA) for 30 min. Tumor cell number was adjusted to 1 × 10^5^ cells/ml in PBS or blood from healthy human volunteers and the reaction tube completely filled. A cmHsp70.1 mAb-coated detector (kindly provided by GILUPI GmbH, Potsdam, Germany) was placed in a silicone stopper and incubated with the cell suspension for 30 min under rotation. After four washing steps, unstained cells were fixed with acetone for 30 s and blocked with 3% w/v BSA in PBS for 30 min. Cells were stained with a cocktail of cytokeratin antibodies at RT for 30 min in the dark: CK7 (LP5K, Millipore, Billerica, USA), CK19 (A53-B/A2, Exbio, Vestec, Czech Republic), and panCK (4–6, 8, 10, 13, 18) (C11, Exbio, Vestec, Czech Republic). After a washing step, cell nuclei were stained with Hoechst 33342 (Thermo Fisher Scientific, Waltham, USA) for 5 min at RT. The presence of cells on the CellCollector® detector was analyzed with the Zeiss Axio Imager.M2 or the Axio Observer.Z1 (Zeiss, Jena, Germany). Cells were counted on two sides of the wire, after a rotation of 180°. Image analysis and processing was performed using AxioVision SE64 Rel. 4.9. For the transfer of captured cells back into cell culture, the tip of an unfixed detector was cut off and incubated in a flask with the appropriate cell culture medium.

## Results

### mHsp70 and EpCAM expression varies in different tumor cell types, but remains stable after TGFβ- and L-lactic- acid-induced EMT

Hsp70 is frequently expressed on the membrane of primary tumor cells from a variety of different entities (32–34, 36), and expression has been shown to be increased by radio(chemo)therapy and to be of a much higher density on distant metastases (31, 36). As EpCAM is currently the most widely used cell surface marker for antibody-based isolation techniques of CTCs, herein we compared mHsp70 and EpCAM expression by tumor cell lines derived from different entities including breast, lung, melanoma, pancreas, cervix, colon, brain, and squamous cell carcinoma of the head and neck by flow cytometry.

**Figure 1:**
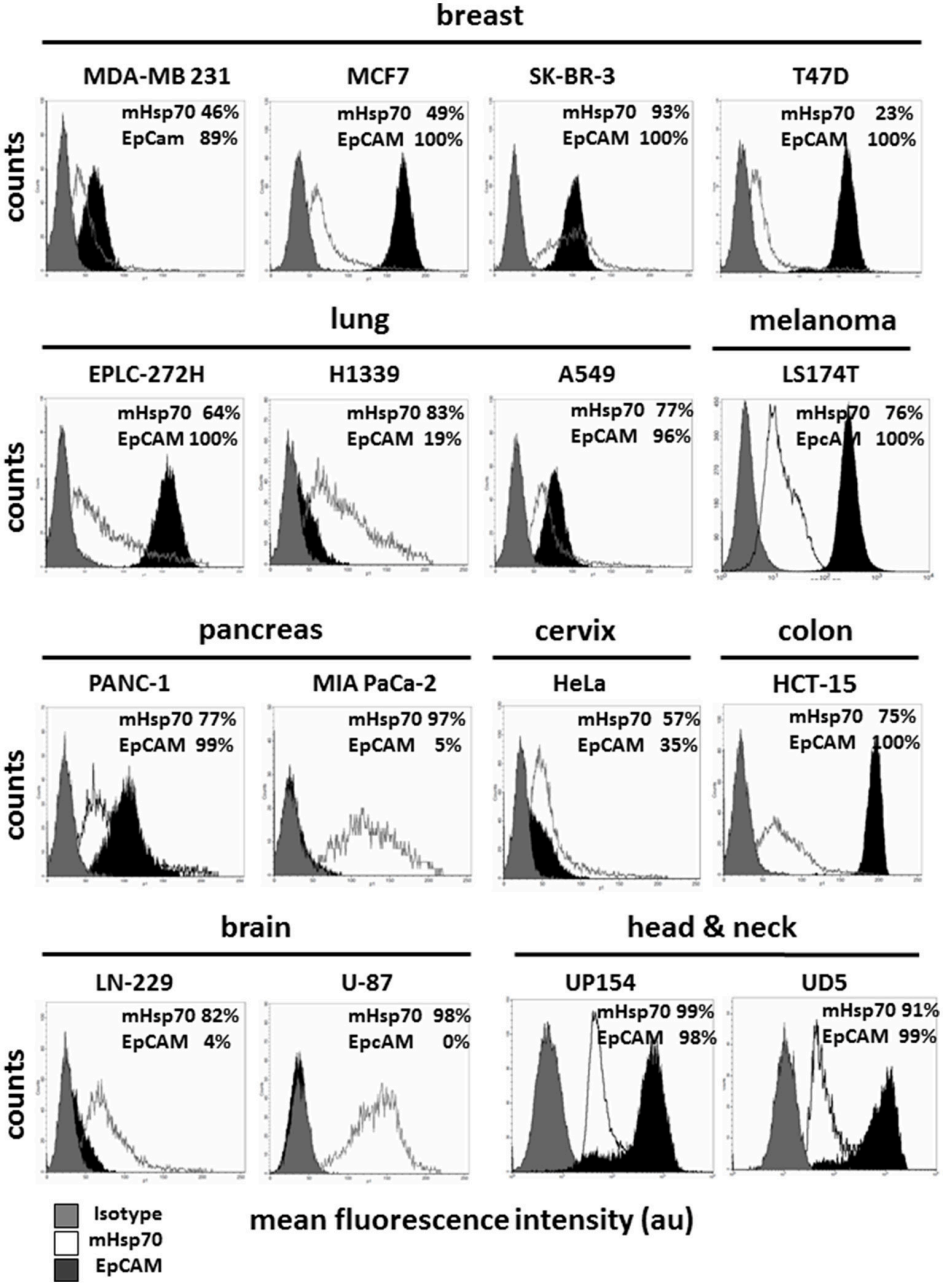
Membrane Hsp70 and EpCAM expression by different cancer cell lines. Cell lines from different cancer entities (breast, lung, melanoma, pancreas, cervix, colon, brain, head, and neck) were screened for their expression of mHsp70 and EpCAM using flow cytometry. Gray histograms represent isotype control, white histograms mHsp70 staining and black histograms EpCAM staining. The numbers in the histograms indicate the proportion of cells stained positively for mHsp70 and EpCAM. The data show one representative result of at least three independent experiments.

As shown in Figure 1, the LN-229 (mHsp70: 80 ± 4%), U-87 (mHsp70: 96 ± 4%) brain, and MIA PaCa-2 (mHsp70: 97 ± 3%) pancreas tumor cells are only positive for mHsp70, but negative for EpCAM. Within the breast cancer cell lines, which are epithelial cells derived from metastatic sites, MDA-MB-231 cells (basal ER^−^ PR^−^ HER2^−^), MCF7 (luminal ER^+^ PR^+^ HER2^−^), and T47D (luminal ER^+^ PR^+^ HER2^−^) exhibited a lower expression of mHsp70 compared to EpCAM, whereas SK-BR-3 cells (luminal ER^−^ PR^−^ HER2^+^) expressed a high expression intensity of both markers, mHsp70 and EpCAM (mHsp70: 95 ± 4%, EpCAM: 96 ± 7%). The patterns of mHsp70 and EpCAM expression by MDA-MB-231 (mHsp70: 50 ± 3%, EpCAM: 90 ± 2%), MCF7 (mHsp70: 51 ± 6%, EpCAM: 99 ± 4%), and T47D (mHsp70: 25 ± 7%, EpCAM: 98 ± 5%) breast cancer cells that were analyzed at TUM and NTU were nearly identical (data not shown).

Heterogeneities in the expression pattern of mHsp70 and EpCAM were also observed for human lung (EPLC-272-H, H1339, A549), melanoma (LS174T), pancreatic (PANC-1, MIA PaCa-2), cervix (HeLa), colon (HCT-15), brain (LN-229, U-87), and head and neck (UP154, UD5) cancer cell lines (Figure 1).

**Figure 2:**
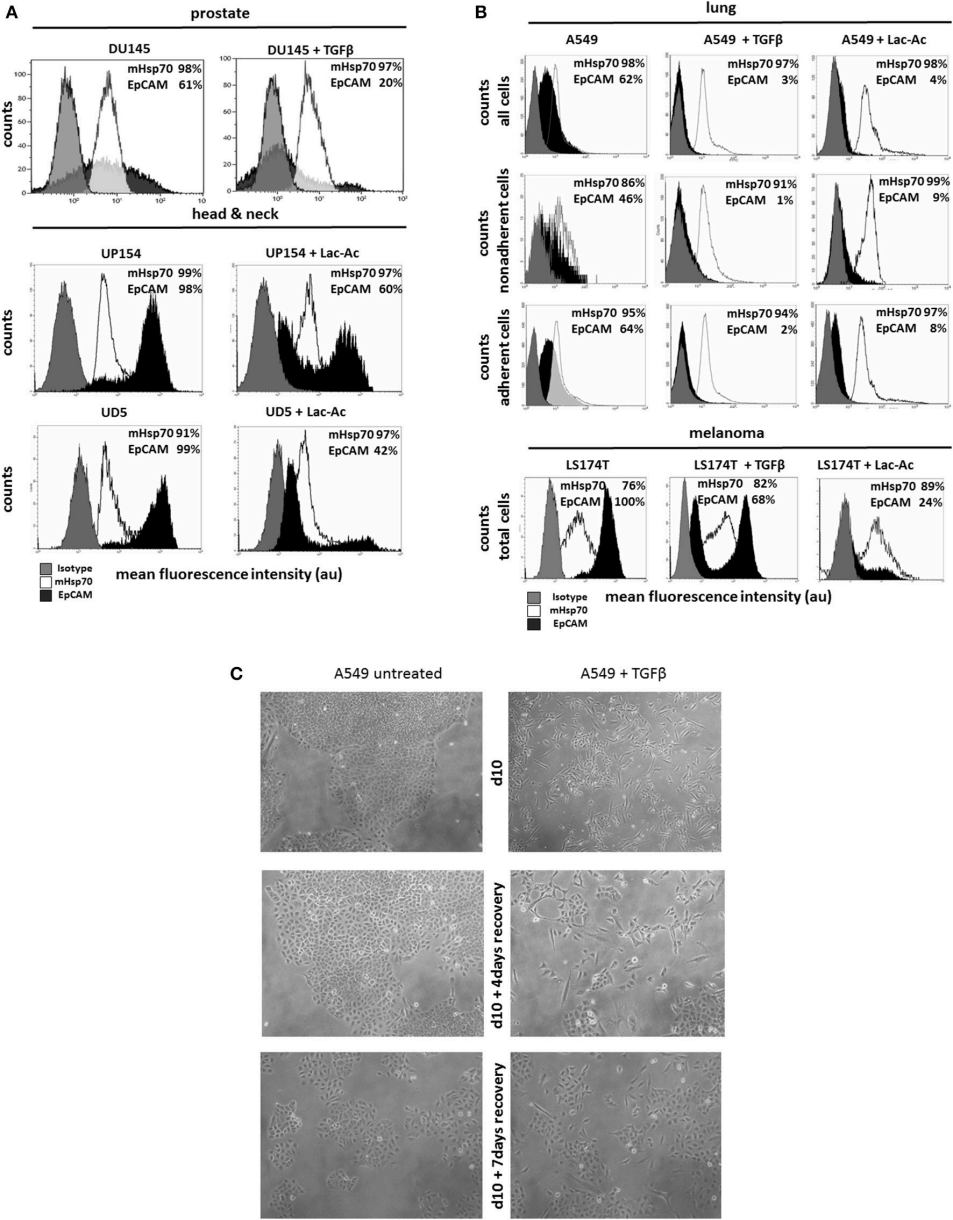
Influence of TGFβ- and L-lactic-acid (Lac-Ac)-induced EMT on mHsp70 and EpCAM expression by DU145 (prostate), UP154 (head and neck), UD5 (head and neck), A549 (lung) cancer cells, and LS174T melanoma cells. **(A)** DU145 cells were cultured with TGFβ for 10 days, UP154 and UD5 cells were cultured for 2 days with L-lactic-acid (Lac-Ac), after which the expression of mHsp70 and EpCAM was determined. **(B)** A549 and LS174T cells were cultured either with TGFβ for 10 days or with L-lactic-acid (Lac-Ac) for 2 days, after which the expression of mHsp70 and EpCAM was determined on viable cells using flow cytometry. Moreover, non-adherent and adherent cells were analyzed separately in A549 cells for their mHsp70 and EpCAM expression. Gray histograms represent isotype control, white histograms mHsp70 staining and black histograms EpCAM staining. The proportion of cells stained positively for mHsp70 and EpCAM are indicated in each histogram. The data show one representative result of at least three independent experiments. **(C)** Representative photomicrographs of untreated and TGFβ treated A549 cells on day 10, day 10 + 4 days recovery without TGFβ and day 10 + 7 days recovery without TGFβ.

In order to simulate EMT *in vitro*, prostate (DU145), head and neck (UP154, UD5) and lung (A549) cancer cells and malignant melanoma (LS174T) cells were incubated with TGFβ for 10 days and with L-lactic-acid (Lac-Ac, 10 mM, pH 6.8) for 2 days. Although TGFβ treatment drastically reduced the expression of EpCAM by DU145 cells (64 ± 5% to 18 ± 1%) (Figure 2A) and A549 (62 ± 1% to 2 ± 1%) cells (Figure 2B), mHsp70 expression was retained at nearly 100% after the induction of EMT in both cell lines (Figures 2A,B). Similar results were observed after a treatment of UP154 and UD5 head and neck cancer cells with TGFβ for 10 days (data not shown) and L-lactic-acid for 2 days (Figure 2A).

A comparative analysis of the mHsp70 and EpCAM expression on non-adherent and adherent A549 (Figure 2B) cells revealed a higher expression of EpCAM on the adherent, epithelial-like cell population (A549, EpCAM: 64%) compared to the non-adherent, mesenchymal-like (A549, EpCAM: 46%) tumor subpopulation that dropped significantly upon treatment with TGFβ and L-lactic-acid (Figure 2B). Concomitant with the mesenchymal transition induced by TGFβ and L-lactic-acid, the number of viable, non-adherent cells in the A549 cultures increased 15- and 11-fold, respectively. Similar results were observed for the LS174T cells. Following treatment with TGFβ and L-lactic-acid, the proportion of EpCAM positive cells decreased from 100 to 68% and 24%, respectively, whereas the proportion of cells expressing mHsp70 slightly increased from 76 to 82% and 89%, respectively (Figure 2B).

Representative photomicrographs illustrating morphological changes induced by treating A549 cells with TGFβ for 10 days followed by a recovery period in the absence of TGFβ for 0 (d10), 4 (d10 + 4 days recovery), and 7 (d10 + 7 days recovery) days are provided in Figure 2C. Following treatment with TGFβ, a large proportion of cells detaches from the culture flasks and the remaining adherent cells show a spindle-like phenotype. Within the recovery period of 4 and 7 days, tumor cells became adherent and showed an epithelial-like phenotype. Similar results were observed after treatment with L-lactic-acid (data not shown).

### Recovery of “spiked” tumor cells by magnetic bead-based separation system depends on the proportion of mHsp70 positive cells

To assess and compare the capacity of cmHsp70.1 mAb coated magnetic beads to separate CTCs from buffer and peripheral blood and its relationship with mHsp70 expression, magnetic beads covalently coupled to cmHsp70.1 mAb were incubated with buffer and blood from healthy volunteers that had been “spiked” with identical numbers of SK-BR-3 breast cancer cells (~93% positive for mHsp70 expression) or T47D breast cancer cells (~23% positive for mHsp70) expression for 1 h at 37°C.

**Figure 3:**
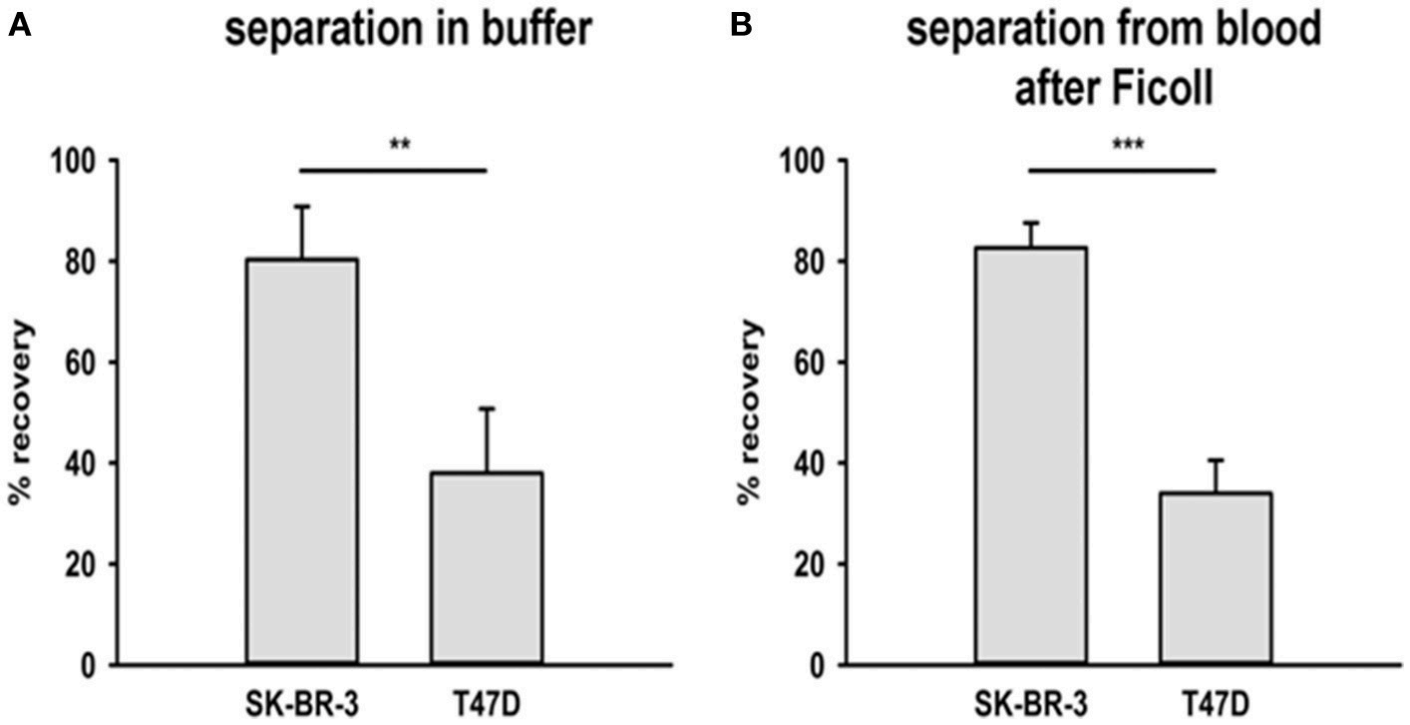
Recovery of tumor cells from buffer and a mixture with EDTA blood. Two breast cancer cell lines were chosen for the recovery experiments with cmHsp70.1 mAb-functionalized magnetic bead system: SK-BR-3 with a high surface expression of Hsp70 and T47D with a low surface expression of Hsp70. **(A)** Tumor cells were spiked into buffer and incubated with the beads for 1 h at 37°C. The percentage of recovered cells aligned with the mHsp70 status of the cells (see Figure 1), as determined by flow cytometry, with 80 ± 11% of SK-BR-3 and 28 ± 13% of T47D cells being recovered. **(B)** Tumor cells were spiked into blood from healthy donors after depletion of erythrocytes and incubated with cmHsp70.1 mAb-functionalized magnetic beads for 1 h at 37°C. Recovery from blood was very similar to that in buffer, with an 83 ± 5% recovery of SK-BR-3 cells and a 34 ± 7% recovery of T47D cells. Data are from at least 3 independent experiments and are expressed as means ± SD.

As shown in Figure 3, following magnetic separation, the recovery of SK-BR-3 cells was significantly greater than the recovery of T47D cells (mHsp70: 80 ± 11% vs. 38 ± 13% and 83 ± 5% vs. 34 ± 7%, respectively) for both experiments (*n* = 3) in buffer (Figure 3A) and blood (Figure 3B). Capture therefore correlated with the percentage of mHsp70 positive cells in the respective tumor cell populations.

### Vybrant™ CFDA-stained and unstained tumor cells can be recovered with cmHsp70.1 mAb-functionalized CellCollector® system

**Figure 4:**
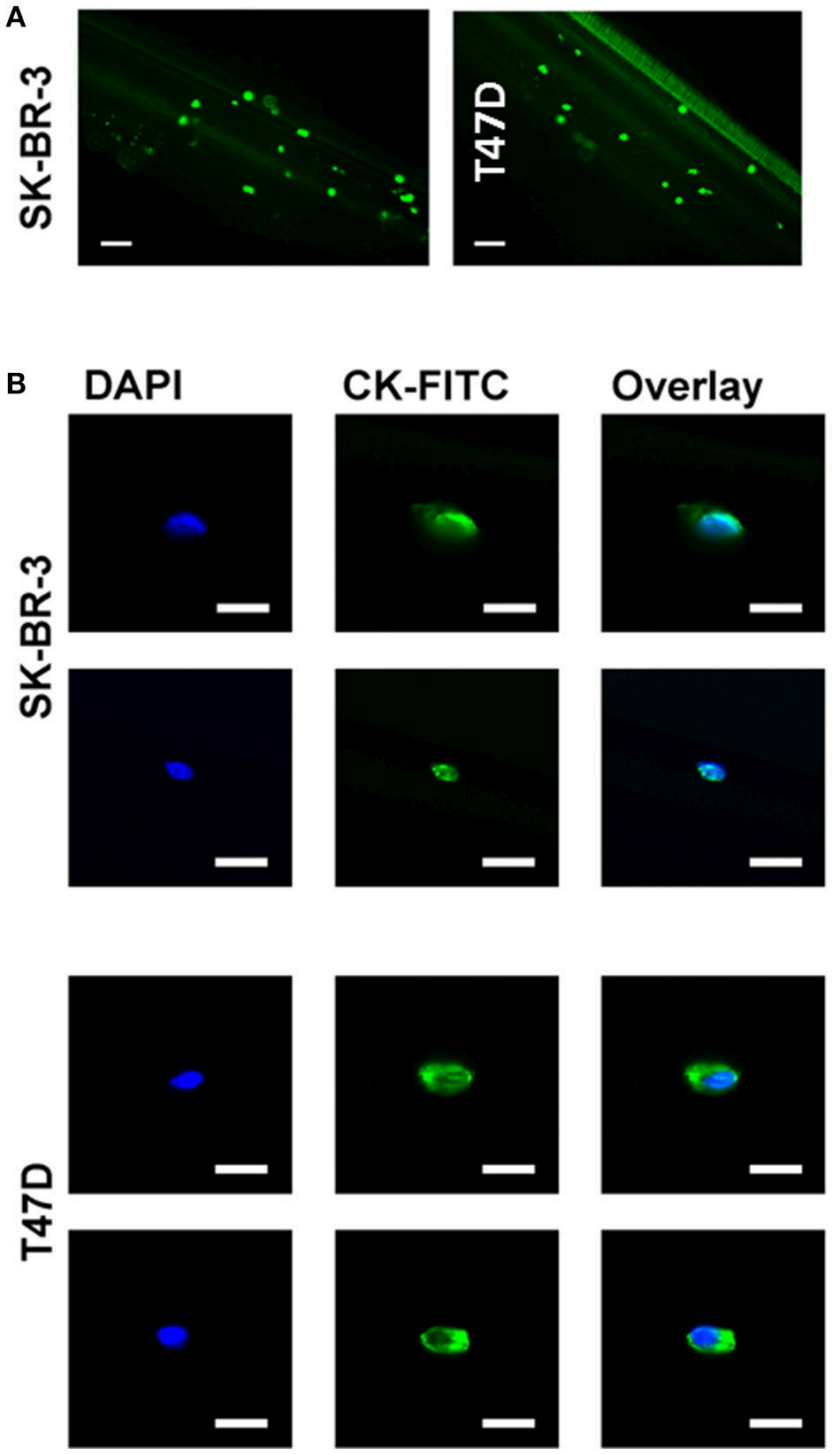
Capture of SK-BR-3 and T47D cells with the cmHsp70.1 mAb-functionalized CellCollector® system. **(A)** SK-BR-3 cells were stained with CFSE (green) and incubated with the cmHsp70.1 mAb-functionalized CellCollector® system. Captured cells were analyzed with a fluorescence microscope. Scale bar 100 μm. **(B)** SK-BR-3 or T47D cells were incubated with the cmHsp70.1 mAb-functionalized CellCollector® system for 30 min, fixed and stained with cytokeratin antibodies (CK-FITC, green) and Hoechst 33342 (DAPI, blue). Scale bar 25 μm.

The primary limitation of most *ex vivo* CTC isolation techniques is the relatively small volume of blood which is used (7.5 ml) and the low numbers of CTCs that can be derived therefrom. The GILUPI CellCollector® directly captures tumor cells from the patient's blood stream using an EpCAM antibody-coated CellCollector® system inserted into the cubital veins over a 30 min period. To evaluate the capacity of adapting this approach to capture cells expressing mHsp70, the cmHsp70.1 mAb was covalently linked to the surface of the detector tip of the CellCollector® system and this was then incubated with a suspension of Vybrant™ CFDA pre-stained or unstained SK-BR-3 or T47D cells in buffer. The capture of cells was then determined using a fluorescence microscope. As shown in Figure 4A, the cmHsp70.1 mAb-functionalized CellCollector® system captured more Vybrant™ CFDA pre-stained SK-BR-3 cells than T47D tumor cells. Similar results were obtained using unstained tumor cells that were subsequently fixed and stained with a cocktail of cytokeratin antibodies directly on the cmHsp70.1 mAb-functionalized CellCollector® system (data not shown). Representative fluorescence micrographs of SK-BR-3 and T47D tumor cells on the cmHsp70.1 mAb-functionalized wires after staining with DAPI and CK-FITC are provided in Figure 4B.

### Tumor cells collected with cmHsp70.1 mAb-functionalized CellCollector® system from the blood maintain their phenotype and can be propagated in cell culture

**Figure 5:**
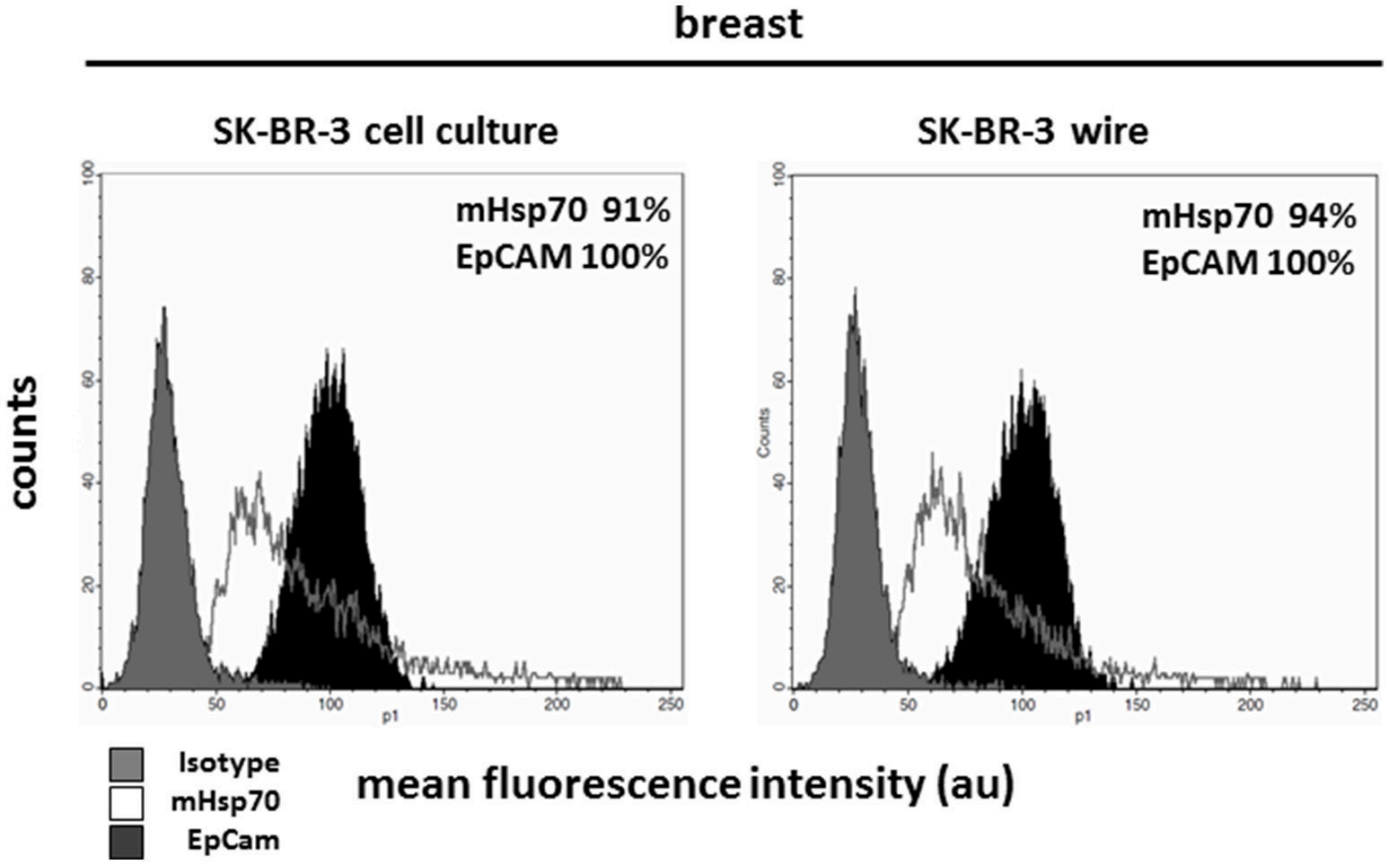
Capture of SK-BR-3 tumor cells with the cmHsp70.1 mAb-functionalized CellCollector® system and subsequent cultivation and analysis. SK-BR-3 cells were captured from a mixture of the tumor cell line with EDTA blood. The functional part of the wire was cut from the detector and transferred to a cell culture flask. Cells that became adherent in the flask were expanded and finally analyzed with flow cytometry. Cells captured with the cmHsp70.1 mAb-functionalized wire (right panel) showed characteristics comparable to those of SK-BR-3 cells from cell culture (left panel). Gray histograms represent isotype control, white histograms mHsp70 staining and black histograms EpCAM staining. The numbers in the histograms indicate the proportion of cells stained positive for mHsp70 and EpCAM. Data are a representative result of at least three independent experiments.

Circulating tumor cells (CTCs) are very rare and further analysis of these might require the *in vitro* expansion of isolated cells. The capacity to transfer cells that have been captured using the cmHsp70.1 mAb-based CellCollector® system into cell culture was therefore evaluated. For this, the cmHsp70.1 mAb-functionalized CellCollector® system was incubated with peripheral blood which had been spiked with unstained SK-BR-3 breast cancer cells under sterile conditions. After washing, the functionalized part of the detector was placed in a cell culture flask with appropriate culture medium. After 24 h at 37°C, SK-BR-3 cells became adherent to the culture flask and started to grow. Flow cytometric analysis revealed that the phenotype of SK-BR-3 cells that were collected using the cmHsp70.1 mAb-based CellCollector® wire system was comparable to that of SK-BR-3 cells in cell culture (mHsp70: 94% vs. 91%; EpCAM: 100% vs. 100%, respectively) (Figure 5).

### cmHsp70.1 mAb-functionalized magnetic beads can isolate CTCs from EDTA blood of patients with squamous cell carcinoma of the head and neck (SCCHN) and non-small cell lung carcinoma (NSCLC)

**Figure 6:**
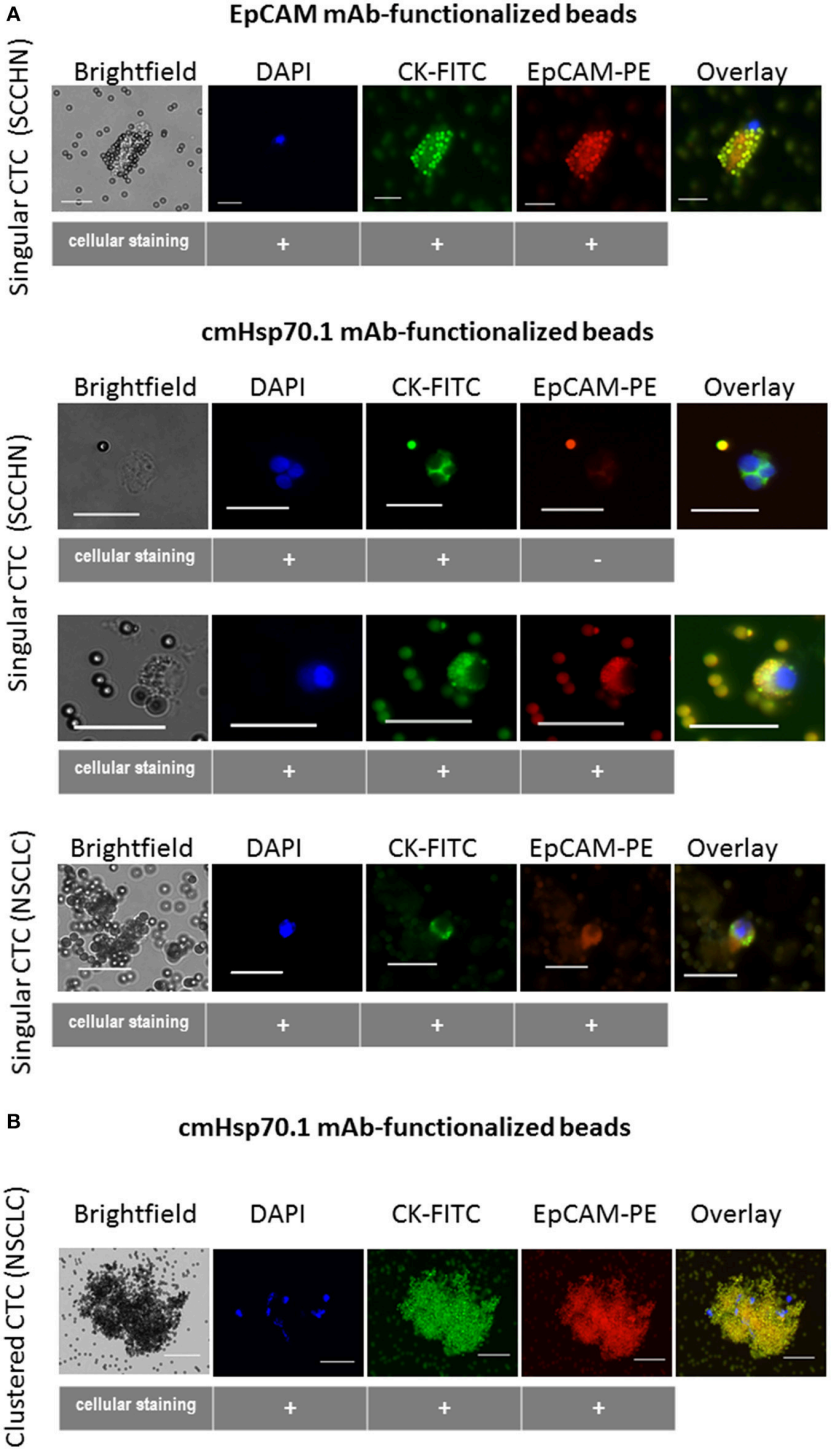
Capture of CTCs from the EDTA blood of patients using EpCAM mAb- or cmHsp70.1 mAb-functionalized magnetic beads. Representative views of singular **(A)** and clustered **(B)** CTCs derived from EDTA blood (7.5 ml, each) of patients with squamous cell carcinoma of the head and neck (SCCHN) and non-small cell lung carcinoma (NSCLC) bound to the EpCAM mAb- and cmHsp70.1 mAb-functionalized beads: brightfield, DAPI (blue), FITC-labeled cytokeratin antibody (CK-FITC, green), PE-labeled EpCAM (EpCAM-PE, red), overlay derived thereof. Scale bar 20 μm.

Given the rarity of CTCs, it is also important to confirm that cmHsp70.1 mAb-functionalized magnetic beads can isolate CTCs from the peripheral blood of patients with cancer. We obtained EDTA blood from 8 patients with SCCHN, 1 patient with CUP tumor of the head and neck, and 3 patients with advanced NSCLC at diagnosis. The age of the SCCHN patients ranged between 51 and 72 years and that of NSCLC patients between 71 and 76. The clinical parameters of all patients are summarized in Table 1. Using magnetic bead separation with cmHsp70.1 mAb-functionalized magnetic beads we succeeded to isolate CTCs from 7 of 8 patients with SCCHN and from 3 of 3 patients with advanced NSCLC. The number of CTCs isolated from 7.5 ml EDTA blood of patients which are shown in Table 1 ranged between 0 and 92 cells. Representative fluorescence microscopic views of singular and clustered CTCs derived from equal amounts of EDTA blood (7.5 ml) of patients with SCCHN and NSCLC isolated with EpCAM mAb and cmHsp70.1 mAb-functionalized beads that were counter-stained with DAPI, CK-FITC, EpCAM-PE are shown in Figures 6A,B. cmHsp70.1 mAb-functionalized bead-separated, cytokeratin-positive CTCs were either EpCAM-positive or EpCAM-negative (Figure 6A). In all tested cases, higher CTC numbers could be isolated from 7.5 ml EDTA blood of patients with SCCHN and NSCLC after separation with cmHsp70.1 mAb-functionalized magnetic beads compared to that isolated with EpCAM mAb-functionalized beads (Table 1). Growth of isolated CTC clones was observed 14 days after limiting dilution cloning of primary CTCs in 10 of 11 patients with SCCHN and NSCLC.

**Table 1:** Clinical characteristics of patients with squamous cell carcinoma of the head and neck (SCCHN), CUP tumor of the head and neck, and non-small cell lung carcinoma (NSCLC) assessed for CTC isolation using cmHsp70.1 mAb- and EpCAM mAb-functionalized magnetic beads.

**ID**	**Location**	**T**	**N**	**M**	**ECE**	**p16**	**Grading**	**CTCs**	**CTCs**
								**cmHsp70.1**	**EpCAM**
**SCCHN**									
1	Larynx	pT3	pN2c	cM0	negative	nd	2	nd	nd
2	Nose	pT1	cN0	cM0	nd	nd	2	0	nd
3	Nose	pT2	cN0	cM0	nd	nd	2	46	nd
4	Tonsils	pT2	pN1	cM0	positive	positive	2	17	nd
5	Hypopharynx	pT2	pN3b	cM0	positive	nd	2	92	nd
6	Oropharynx	pT1	pN0	cM0	negative	positive	2	43	29
7	Mouth	pT1	pN0	cM0	negative	nd	1	9	4
8	Mouth	cT3	pN2	cM0	nd	nd	2	12	nd
9	CUP	cT0	pN3b	cM0	positive	negative	3	32	nd
**ID**	**Location**	**T**	**N**	**M**	**stage**	**PD-L1**		**CTCs**	**CTCs**
	**Lung lobe**							**cmHsp70.1**	**EpCAM**
**NSCLC**									
1	Upper right	cT1a	cN2	cM1c	IVB	20%		18	7
2	Upper right	cT4	cN2	cM1c	IVB	90%		45	nd
3	Upper right	cT3	cN2	cM1a	IVA	30%		35	nd

*Patient code (ID), tumor location, TNM stage, extracellular extension (ECE), p16 status, grading, PD-L1 positivity, and CTC counts. CTCs were isolated either with cmHsp70.1 mAb- and EpCAM mAb-functionalized beads from 7.5 ml EDTA blood*.

Future studies are planned to analyze the molecular characteristics of mHsp70 positive CTCs in a larger cohort of patients with different tumor stages, before therapy and in the follow-up period.

## Discussion

Tumor heterogeneity presents a significant barrier to the effective treatment of cancers in general and aggressive, therapy-resistant metastatic cancers particularly. Circulating tumor cells (CTCs) present in the peripheral blood offer an invaluable liquid biopsy-based approach for interrogating and categorizing disease, evaluating prognosis and monitoring therapeutic responsiveness in patients with different cancer entities. Crucially, as the number of CTCs correlates with the stage of metastasis and is inversely related to therapeutic outcome, determining the presence, and interrogating the biology of CTCs provide the insights into the development and mechanism(s) of metastatic spread (39) and therapeutic resistance. Both are essential if progress in reducing cancer mortality is to be made, given that 90% of cancer-related deaths are due to therapy-resistant metastatic disease rather than the primary tumor (40). The ability to phenotypically and genetically profile CTCs from individual patients would provide an unprecedented insight into the biology of the disease on an individual basis and would inform and underpin the more effective delivery of precision-based, individualized medicine.

Currently, existing antibody-based approaches for isolating CTCs use magnetic beads or particles for the capture and isolation of CTCs. Cell-Search®, the current FDA-approved “gold standard” (22) and the GILUPI CellCollector® (28) rely on the cell surface expression of EpCAM (CD326) on the CTCs that are to be captured (41). However, the expression of EpCAM is often downregulated on CTCs (2), and is also known to be downregulated following EMT, a process which is integral to the transition of adherent tumor cells to a migratory status which enables them to depart from the primary tumor, enter the circulation and seed distal sites (2). As cells that have undergone EMT are those that are mostly involved in the establishment and progression of metastatic disease, it is essential that strategies for detecting, isolating and subsequently characterizing these CTCs are based on approaches that can best capture these cells. It is therefore essential that tumor markers that are expressed on CTCs before and after EMT are used as targets for capturing CTCs.

The search for universal tumor markers has revealed that the major stress-inducible Hsp70 is frequently expressed on the plasma membrane of a wide variety of tumor entities, but not non-transformed cells and tissues (31–33). It has also been demonstrated that metastases often express higher levels of mHsp70 compared to the primary tumor (33–36). It is therefore likely that CTCs will preferentially express mHsp70 over EpCAM.

We have previously reported on the development and validation of a mouse monoclonal antibody (cmHsp70.1) which is able to bind to this membrane form of Hsp70 (37). The unique binding characteristics of this antibody make it an ideal candidate for the development of new bead- or wire-based approaches for capturing CTCs that will not be detected using EpCAM-based approaches, due to the downregulation of EpCAM expression after EMT. Herein, we profiled the expression of mHsp70 and EpCAM by cancer cell lines derived from a range of different tumor entities and the influence of TGFβ- and L-lactic-acid-induced EMT on expression and examined the capacity of bead- and wire-based approaches for capturing cancer cells differentially expressing mHsp70.

Flow cytometric profiling revealed heterogeneous mHsp70 and EpCAM expression patterns in cancer cell lines, and it is expected that the level of heterogeneity within and between different tumor entities will be even more marked in the clinical setting. Furthermore, we have demonstrated that although TGFβ- and L-lactic-acid-induced EMT results in a loss of EpCAM expression, mHsp70 expression is retained. The recovery of cancer cell lines expressing high and low levels of mHsp70 from buffer and blood obtained from health donors into which cancer cell lines had been “spiked” using cmHsp70.1 mAb-functionalized magnetic beads and a modified version of the GILUPI CellCollector® incorporating cmHsp70.1 mAb-mediated capture revealed that, as expected, the yield of recovered cells closely correlated with the level of mHsp70 expression of the respective cell line.

Since CTCs are typically very rare−1 ml of peripheral blood of patients with metastatic cancer contains <10 CTCs (9–11, 42)–further analysis and experimentation requires an ability to expand isolated cells *in vitro* (43). We could show that it is possible for CTCs isolated from patients with SCCHN and NSCLC to be transferred into cell culture after cmHsp70.1 mAb-mediated capture. Patient-derived primary CTCs isolated by cmHsp70.1 mAb-functionalized magnetic beads were either EpCAM-negative or EpCAM-positive, indicating that a proportion of EpCAM-negative CTCs cannot be captured using EpCAM-based isolation methods.

In summary, herein we have shown that cmHsp70.1 mAb-functionalized bead- and wire-based approaches provide a promising strategy for the detection and isolation of CTCs from the blood of patients with cancer. This is especially pertinent and important for the capture of CTCs that have undergone EMT, as EMT is associated with a downregulation of EpCAM, the target antigen on which most antibody-based approaches for capturing CTCs currently depend. This new approach could therefore improve the capacity to interrogate the presence and biology of CTCs and form the basis of a new liquid biopsy-based strategy for categorizing disease, evaluating prognosis and for predicting and monitoring therapeutic responsiveness across a range of aggressive cancers.

## Author contributions

SB, SS, CW, WS, DL, GAF, and SW performed experiments. GM and AGP designed study and wrote the manuscript. AP and KK provided samples and clinical data from patients with SCCHN and NSCLC. GP collected, processed, and analyzed samples.

### Conflict of interest statement

The authors AGP and GM declare a potential conflict of interest with respect to the research, authorship, and publication of this article as a consequence of their involvement with multimmune GmbH, München as Chief Executive Officer (APG) and Founder/Chief Scientific Officer (GM). The authors declare that the research was conducted in the absence of any commercial or financial relationships that could be construed as a potential conflict of interest.

## Abbreviations

CK: cytokeratin
CTC(s): circulating tumor cell(s)
EMT: epithelial-to-mesenchymal transition
EpCAM: epithelial cell adhesion molecule
Hsp70: heat shock protein 70
mAb: monoclonal antibody
mHsp70: membrane-bound form of Hsp70
